# Modeling Large Vessel Occlusion Stroke for the Evaluation of Endovascular Therapy According to Thrombus Composition

**DOI:** 10.3389/fneur.2021.815814

**Published:** 2022-01-27

**Authors:** Aurélien Freiherr von Seckendorff, François Delvoye, Paul Levant, Mialitiana Solo Nomenjanahary, Véronique Ollivier, Marie-Charlotte Bourrienne, Lucas Di Meglio, Michel Piotin, Simon Escalard, Benjamin Maier, Solène Hebert, Stanislas Smajda, Hocine Redjem, Mikael Mazighi, Raphael Blanc, Benoit Ho-Tin-Noé, Jean-Philippe Désilles

**Affiliations:** ^1^Interventional Neuroradiology Department, Biological Resource Center, Hôpital Fondation Adolphe de Rothschild, Paris, France; ^2^Université de Paris, Laboratory of Vascular Translational Science, U1148 Institut National de la Santé et de la Recherche Médicale (INSERM), Paris, France

**Keywords:** endovascular therapy (EVT), ischemic stroke, pathophysiology, thrombectomy simulation, thrombus, large vessel occlusion (LVO)

## Abstract

More than 40% of endovascular therapy (EVT) fail to achieve complete reperfusion of the territory of the occluded artery in patients with acute ischemic stroke (AIS). Understanding factors influencing EVT could help overcome its limitations. Our objective was to study the impact of thrombus cell composition on EVT procedures, using a simulation system for modeling thrombus-induced large vessel occlusion (LVO) in flow conditions. In an open comparative trial, we analyzed the behavior of size-standardized platelet-rich and red blood cells (RBC)-rich thrombi during simulated stent retriever-mediated EVT procedures. Sixteen simulated EVT procedures were performed (8 RBC- vs. 8 platelet-rich thrombi). Platelet-rich thrombi were associated with a higher number of stent retriever passes (*p* = 0.03) and a longer procedure duration (*p* = 0.02) compared to RBC-rich thrombi. Conversely, RBC-rich thrombi released more embolic fragments than platelet-rich thrombi (*p* = 0.004). Both RBC-rich and platelet-rich thrombi underwent drastic compaction after being injected into the *in vitro* circulation model, and histologic analyses showed that these EVT-retrieved thrombi displayed features comparable to those previously observed in thrombi from patients with AIS patients having LVO, including a marked structural dichotomy between RBC- and platelet-rich areas. Our results show that the injection of *in vitro*-produced thrombi in artificial cerebrovascular arterial networks is suitable for testing recanalization efficacy and the risk of embolization of EVT devices and strategies in association with thrombus cell composition.

## Introduction

Endovascular therapy (EVT) has dramatically improved the rate of early recanalization and is now the standard-of-care for patients with acute ischemic stroke (AIS) and anterior circulation of large vessel occlusion (LVO) (1). However, irrespective of the first approach strategy used for EVT, more than 40% of EVT do not achieve complete reperfusion, which is defined as the final modified thrombolysis in cerebral infarction (mTICI) score of 2C or 3 (2, 3). Moreover, complete reperfusion after a single EVT pass (first pass effect), which is associated with the best clinical outcome, occurs in only 25% of EVT-treated patients (4). Thus, there is a need and room for the development of new devices and strategies to improve the removal of culprit thrombi and the outcome of EVT-treated patients with AIS.

Several *in vitro* models have been developed to assess the safety and effectiveness of EVT strategies (5–9). These models are based on the use of artificial cerebrovascular arterial networks made of polymer tubes, in which the thrombi generated from the human or animal blood are injected under flow. Most of the studies using these models aimed at comparing the efficiency of various EVT devices and strategies (e.g., stent retriever under continuous aspiration vs. contact aspiration alone) to retrieve thrombus analogs. Remarkably, although various associations between thrombus composition and per-procedural and clinical outcomes have been reported (10), it is only until recently that these *in vitro* models have been taken advantage of to study the impact of thrombus composition on the EVT procedure. We and others have shown that AIS thrombi are highly heterogeneous in composition, with variable proportions of red blood cells (RBCs), platelets, white blood cells (WBCs), fibrin (ogen), von Willebrand Factor, and extracellular DNA networks (11–14). Using neurovascular thrombectomy simulators, Madjidyar et al. have provided experimental evidence and suggested that retrieval of RBC- and fibrin-richness might be achieved more effectively using, direct aspiration and a stent retriever, respectively, with a balloon guide catheter (8), and Johnson et al. have shown that platelet-retracted thrombi have an increased stiffness that impairs their retrieval by both aspiration and stent retriever (9). While these experimental studies have paved the way for investigating the relationships between thrombus composition and EVT outcome, this opening field of research could benefit from the characterization of thrombus analogs and standardization of their production.

In the present study, we compared the behavior of size- and composition-standardized platelet- and RBC-rich human blood thrombi in an *in vitro* and in flow model of EVT based on the use of a full-scale 3D printing arterial network reproducing the cerebral vascular anatomy and characterized them by immunohistological analysis.

## Materials and Methods

### *In vitro* Human Thrombus Formation

Venous blood samples were collected on citrated tubes (BD Vacutainer, sodium citrate 3.2%) from healthy and fully informed volunteers. Thrombi were generated in an 8-mm-internal-diameter PVC tubing by adding calcium (CaCl_2_, 20 mmol/l) and bovine thrombin (Dade® Thrombin Reagent, Siemens, PA, USA, 1 NIH/ml) to 10 ml of platelet-high/RBC-low or RBC high suspensions in plasma. The clotting mixtures were incubated for 48 h at 37°C and then stored at +4°C for up to 48 h before handling, in order to stop the clotting and to limit the fibrinolytic process. Thrombi were removed from the tubing and 10-mm-long pieces were cut for injection.

Platelet-high/RBC-low suspensions were obtained by mixing 1 part (v/v) of the buffy coat with 6 parts (v/v) of platelet-rich plasma (PRP). RBC-high suspensions were obtained by mixing 3 parts (v/v) of the buffy coat with 4 parts (v/v) of PRP.

The PRP and buffy coat solutions were obtained by centrifuging blood samples at 120 *g* for 15 min at 20°C.

### The EVIAS Plus ^TM^ System

The EVIAS Plus ^TM^ System (Biomodex, Paris, France) is composed of a plug-and-play station to be used in combination with cartridges that provide realistic haptic feedback during the procedure. The proprietary technology, INVIVOTECH®, reproduces the biomechanical behavior of the anatomy of interest by combining soft and rigid polymers in a 3D printing process, based on cerebral artery imaging of a patient. The technology is compatible with X-ray and contrast agent.

A built-in diaphragm pump simulates a heated fluid flow of saline solution through the intracranial cartridge of approximately 120 ml/min, at a stable temperature of 37°C.

**Figure 1:**
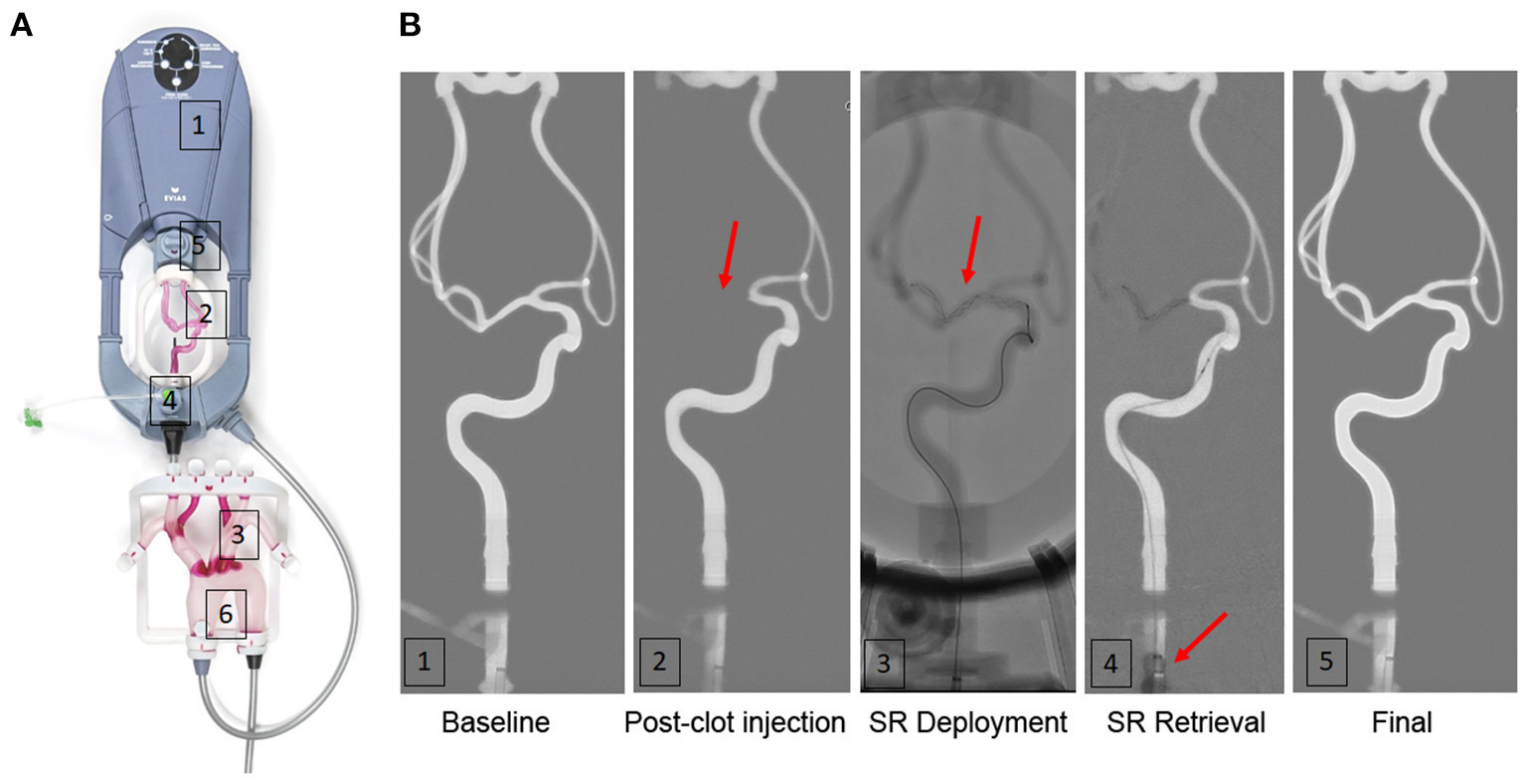
A simulation model for thrombectomy of in flow-injected human thrombi. **(A)** Overview of the EVIAS PlusTM System. (1) a plug-and-play station pump (2) an intracranial artery cartridge; (3) an aortic arch; (4) a thrombus injection system; (5) a distal thrombus filter (700 μm mesh); (6) fluid pressure monitoring outlet. **(B)** processed fluoroscopy imaging of one simulated endovascular therapy (EVT) showing baseline (1), immediately after in flow thrombus injection with M1 occlusion (red arrow) (2), after the stent-retriever deployment (3), during stent-retriever retrieval after proximal balloon inflation (red arrow) (4) and final control (5). SR, stent retriever.

An aortic arch is plugged in front of the station and it allows for more realistic navigation (Figure 1A). It is also irrigated by the fluid flow from the station.

A thrombus injection system integrated into the station enables thrombus insertion inside the intracranial cartridge from the proximal end of the internal carotid artery. The thrombus is then further embolized distally by the fluid flow until it gets impacted in the neurovascular anatomy.

A distal thrombus filter placed behind the intracranial cartridge allows for the collection of any thrombus residues that might have been distally transported during the EVT procedure. The mesh size measures 0.7 mm.

### Experimental Protocol

The EVT simulations were carried out by an experienced neuro interventionalist (7 years of experience, with approximately 50 thrombectomy/aspiration maneuvers per year). Procedures were realized in a dedicated neuro-angiography suite (Azurion, Philips, Netherlands).

Two FDA-approved stent retriever device designs were selected: Solitaire 2 4 × 20 mm or 6 × 30 mm (Medtronic, California, USA) and Trevo XP ProVue 4 × 20 mm or 6 × 25 mm (Stryker, Michigan, USA). A Velocity microcatheter (Penumbra, NY, USA) was used to deliver the stent retriever, a Merci 9F balloon guide catheter (Stryker Michigan, USA) was used to achieve the flow arrest during the stent-retriever retrieval.

Intracranial baseline fluoroscopy imaging was initially done to confirm the perfect patency of the intracranial vessels (Figure 1B). A 10-mm-long thrombus was embolized into the carotid artery from the thrombus injection system placed before the intracranial cartridge (Figure 1A).

About 15 min after thrombus embolus, a second fluoroscopy imaging sequence was taken to assess the level of the intracranial occlusion (MCA-M1 occlusion or ICA T-occlusion).

Next, after the intracranial navigation of a microcatheter under fluoroscopy, a stent retriever was deployed next to the thrombus. After 3 min and using the inflation of the proximal balloon to stop the flow, the stent retriever was removed and a control imaging was acquired to assess the efficacy of the stent-retriever pass.

In case of incomplete recanalization, a maximum of 6 EVT passes were allowed with the possibility to use a distal aspiration catheter after the third pass.

### Outcomes Definition

**Figure 2:**
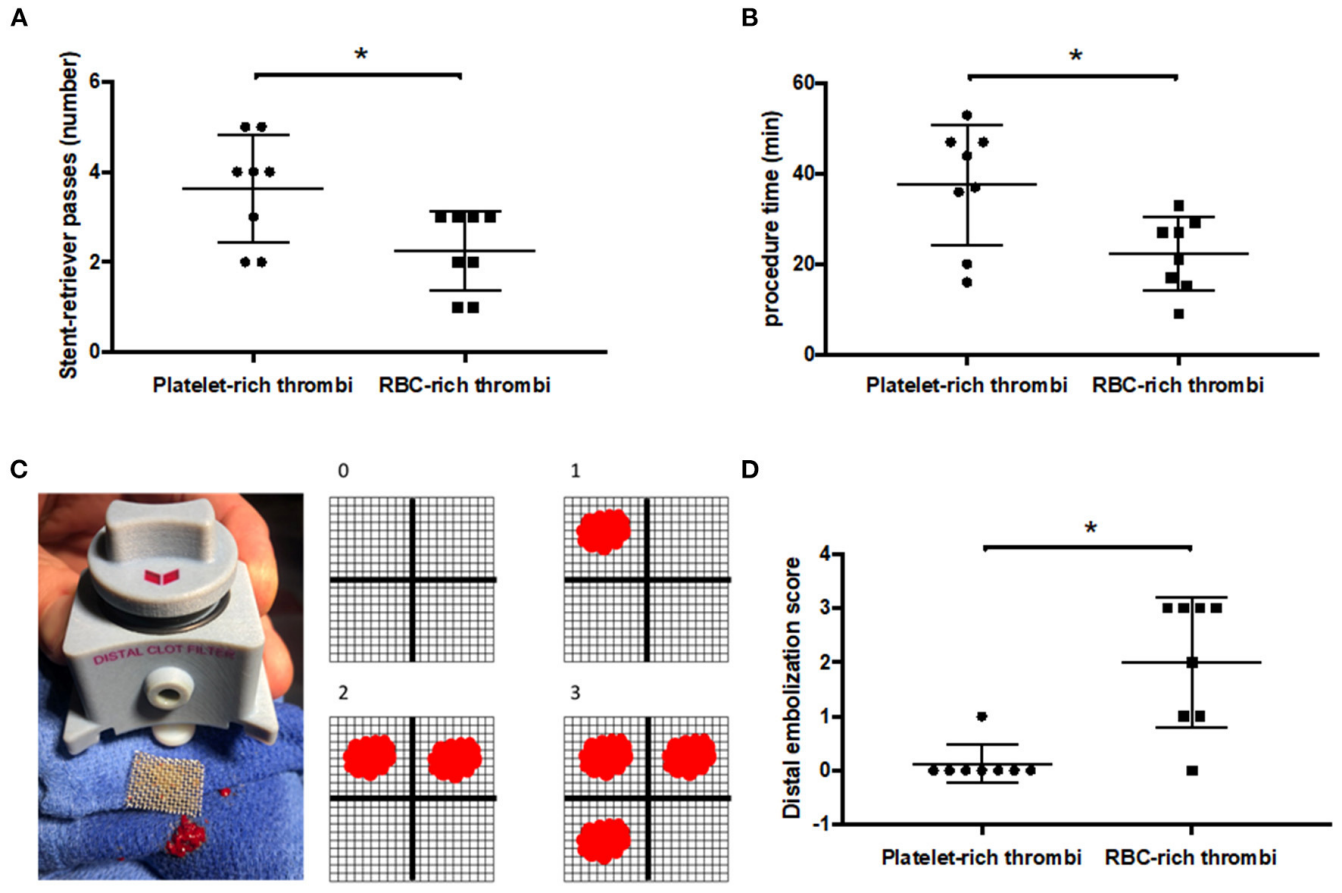
Platelet-rich thrombi require more stent-retriever passes to achieve complete recanalization but release less distal emboli compared to RBC-rich thrombi. **(A)** Number of stent-retriever passes to achieve complete recanalization according to the thrombus composition (platelet and red blood cell (RBC)-rich thrombi). **(B)** Duration of the procedure to achieve complete recanalization. **(C)** Illustration of the distal thrombus filter with its removable mesh of 0.7 mm struts (left panel) and the illustration of the distal embolization score (right panel). **(D)** Distal embolization score according to the thrombus composition (a higher score indicating more distal emboli). *p < 0.05.

The primary assessment criterion was the number of stent-retriever passes to achieve complete recanalization. Other assessment criteria included the EVT procedure duration defined as the delay (in minutes) between the second and final fluoroscopy imaging sequences, the number of distal emboli quantified according to a semi-quantitative scale score (from 0 to 4) based on the number of thrombus fragment deposits present in the distal filter membrane at the end of EVT (Figure 2C), and the incidence of per-EVT ENT in the anterior cerebral artery. The first-pass effect was defined by complete reperfusion after a single EVT pass without fragment deposition in the distal filter.

### Histology and Immunostaining

Thrombi before in flow injection and thrombi retrieved by EVT from the simulation station were fixed for 48 h in 3.7% paraformaldehyde (PFA), embedded longitudinally in paraffin, and sectioned at 6 μm. After deparaffinization, antigen retrieval with Tris EDTA pH 9.0 (Target Retrieval Solution, S2367, Dako), and blocking for 1 h with 3% of bovine serum albumin (BSA) in PBS 1X, the tissue sections were incubated with primary antibodies to CD42b (2 μg/ml, IM0409, Beckman Coulter), glycophorin A (10 μg/ml, M0819, Dako), and fibrinogen (10 μg/ml, A0080, Dako) for 2 h at room temperature. After being washed two times in PBS, followed by 1 h of incubation with secondary antibodies, tissue sections were counterstained with Hoechst 33,342 (10 μg/ml, H3570, Life Technologies), and mounted in a fluorescent mounting medium (Dako). Hematoxylin and Eosin (H&E) staining was also performed as described (13). The H and E and fluorescent images were acquired using a Hamamatsu (Japan) nanozoomer slide scanner and a Leica DMi8 microscope, respectively (Leica Microsystems, Wetzlar, Germany), with LAS X Software.

### Statistical Data Analysis

Each thrombus injection tested in the study was considered to be an independent data point, and statistical analyses comparing the two types of thrombi (platelet-rich vs. RBC-rich thrombi) were performed. All values are presented as mean ± SD. For statistical analyses, PrismGraph 4.0 software (GraphPad Software, San Diego, CA) was used and differences between the two groups were assessed using the Mann–Whitney test. Values of *P* < 0.05 were considered statistically significant.

## Results

### Standardization of *in vitro* Thrombus Formation

Two types of blood cell suspensions were selected for this study. The two types of cell suspensions differed mostly in their RBC/platelet ratio and were then designated as platelet- or RBC-rich suspensions. Nevertheless, it is worth noting that, as compared to the whole blood, both types of blood cell suspensions displayed relatively low RBC counts (Table 1). Platelet-rich suspensions had more platelets (432 vs. 300 x 10^3^/μl; *p* < 0.001), less RBC (0.38 vs. 2.01 x 10^6^/μl; *p* < 0.001), and fewer leukocytes (2.88 vs. 6.80 x 10^3^/μl; *p* = 0.07) than RBC-rich suspensions (Table 1). All 16 thrombi prepared from these blood cell suspensions successfully caused an arterial occlusion. An example of occlusion following in flow thrombus injection is shown in Figure 1B and in the Supplementary Movie. There was no difference in occlusion site localization according to the type of thrombus (3 M1 and 5 ICA-T occlusions in each arm).

**Table 1:** Composition of blood cell suspensions for the preparation of red blood cell- or platelet-rich thrombi.

	**Type**	**RBC (10^**6**^/μl)**	**Hemoglobin (g/dl)**	**Hematocrit (%)**	**Platelets (10^**3**^/μl)**	**WBC (10^**3**^/μl)**
Cell suspension 1	RBC-rich	1.82	5.31	18.25	300.94	9.93
Cell suspension 2	RBC-rich	1.99	6.27	20.41	300.02	5.84
Cell suspension 3	RBC-rich	2.21	6.34	21.81	301.32	4.64
Cell suspension 4	Platelet-rich	0.46	1.37	4.28	451.01	3.14
Cell suspension 5	Platelet-rich	0.28	0.91	2.78	437.74	2.78
Cell suspension 6	Platelet-rich	0.39	1.21	3.86	408.07	2.71
**RBC-rich**	Mean	2.01	5.97	20.16	300.76	6.80
	SD	0.19	0.57	1.79	0.67	2.77
**Platelet-rich**	Mean	0.38	1.16	3.64	432.27	2.88
	SD	0.09	0.24	0.77	21.98	0.23

*Red blood cells (RBC)-rich and platelet-rich blood cell suspensions were prepared from three different donors (one RBC-rich and one platelet-rich cell suspension per donor) to produce a total of 16 thrombi, eight from each type of suspension*.

### Thrombus Composition Is Associated With Different EVT Outcomes

All 16 thrombi were successfully retrieved by EVT. Thrombi prepared from platelet-rich suspensions required a statistically significant higher number of stent-retriever passes to obtain complete recanalization (3.6 ± 1.2 vs. 2.2 ± 0.9; *p* = 0.03, Figure 2A). Of note, the use of adjunctive distal aspiration after the third pass was necessary for 5 out of 8 platelet-rich thrombi but in none of the RBC-rich thrombi. As a result, the mean duration of the EVT procedure was longer for the retrieval of platelet-rich thrombi than for that of RBC-rich thrombi (37.5 ± 13.3 vs. 22.2 ± 8.1 min; *p* = 0.02, Figure 2B). Complete recanalization without thrombus fragmentation after a single stent-retriever pass (first pass effect) occurred in 1 out of 8 RBC-rich thrombi but in none of the 8 platelet-rich thrombi. Regarding thrombus fragmentation and embolization during EVT, while 5 out of 8 RBC-rich thrombi released fragments into the anterior cerebral artery during the procedure, only 1 out of 8 platelet-rich thrombi released fragments into the anterior cerebral artery(*p* = 0.12). Accordingly, the mean embolization score reflecting the amount of thrombus material deposited on the output filter membrane was statistically significantly higher in RBC-rich thrombi compared to platelet-rich thrombi (0.12 ± 0.35 vs. 2 ± 0.2, *p* = 0.004, Figure 2D).

### In-Flow Injected Thrombi Display Structural Features of AIS Thrombi

**Figure 3:**
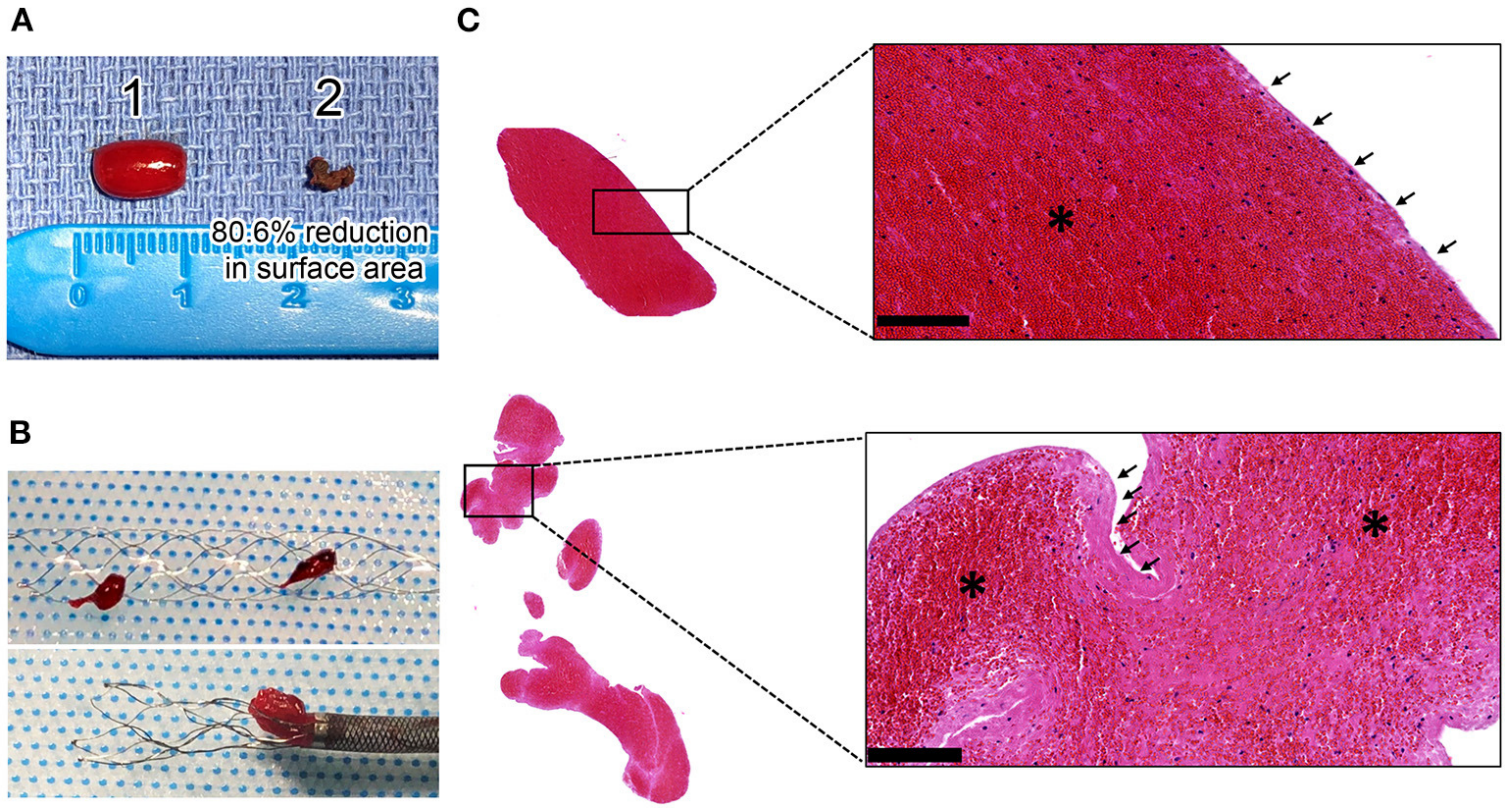
Histological aspect and organization of human thrombi causing large vessel occlusion (LVO) in the simulation station. **(A)** Representative image of a human RBC-rich clot before in flow injection (1) and after (2) its retrieval by EVT. **(B)** Macroscopic view in the stent retriever of the RBC-rich thrombus fragmented into two pieces (top panel) and a platelet-rich thrombus stuck in the aspiration catheter (panel at the bottom) **(C)** Representative H and E staining images of an RBC-rich (top) and platelet-rich (bottom) thrombus at the bottom showing: RBC-rich areas in red (*), fibrin/platelet-rich area in pink (arrows) and leukocytes in purple. Insets show higher magnification views of the squared areas. Bars = 100 μm.

**Figure 4:**
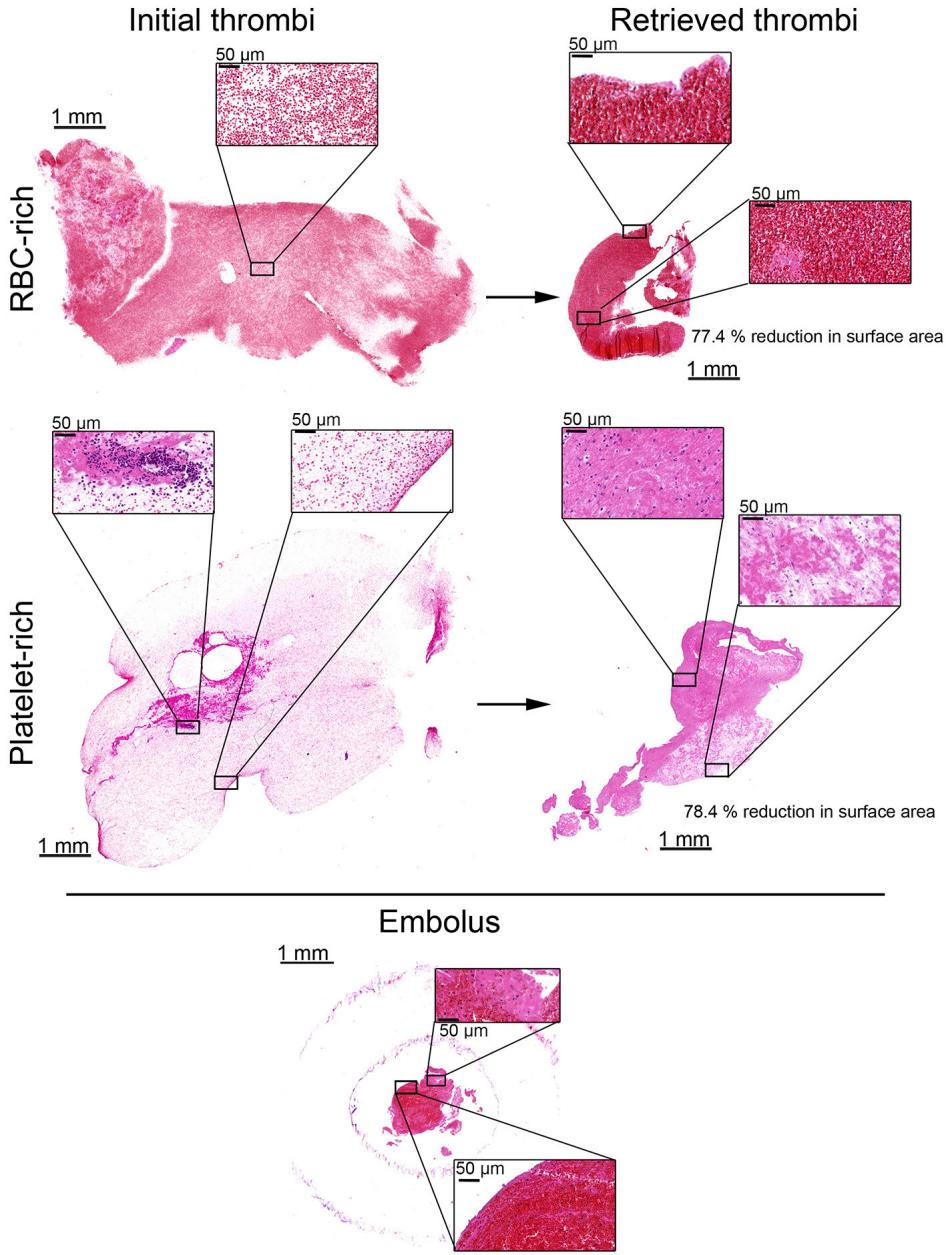
Histological aspect of red blood cell-rich and platelet-rich thrombi before and after injection in the simulation system and EVT procedure. Representative images of hematoxylin and eosin (H and E) stained red blood cell (RBC)-rich (upper panels) and platelet-rich (middle panels) thrombi before being injected into the simulation system (left panels), and after their recovery by EVT (right panels). Insets show higher magnification images of the squared areas. Note that both types of retrieved thrombi are drastically compacted as shown by their reduced size and higher cell density as compared to initial thrombi. The lower panels show H and E images of the RBC-rich thrombus responsible for large vessel occlusion in the intracranial cartridge. Insets show higher magnification images of the squared areas. Note the compaction of thrombus components in both RBC-poor (upper inset) and RBC-rich (lower inset) areas.

Comparative macroscopic views of thrombi before in flow injection and after EVT revealed that, irrespective of their blood cell composition, the retrieved thrombi were highly compact compared to original *in vitro*-generated thrombi (Figures 3A, 4). Importantly, thrombus histology in the intracranial cartridge showed compaction comparable to that of EVT-retrieved thrombi (Figure 4), thus indicating that thrombus compaction was the result of hydrodynamic pressure rather than an EVT-related change.

**Figure 5:**
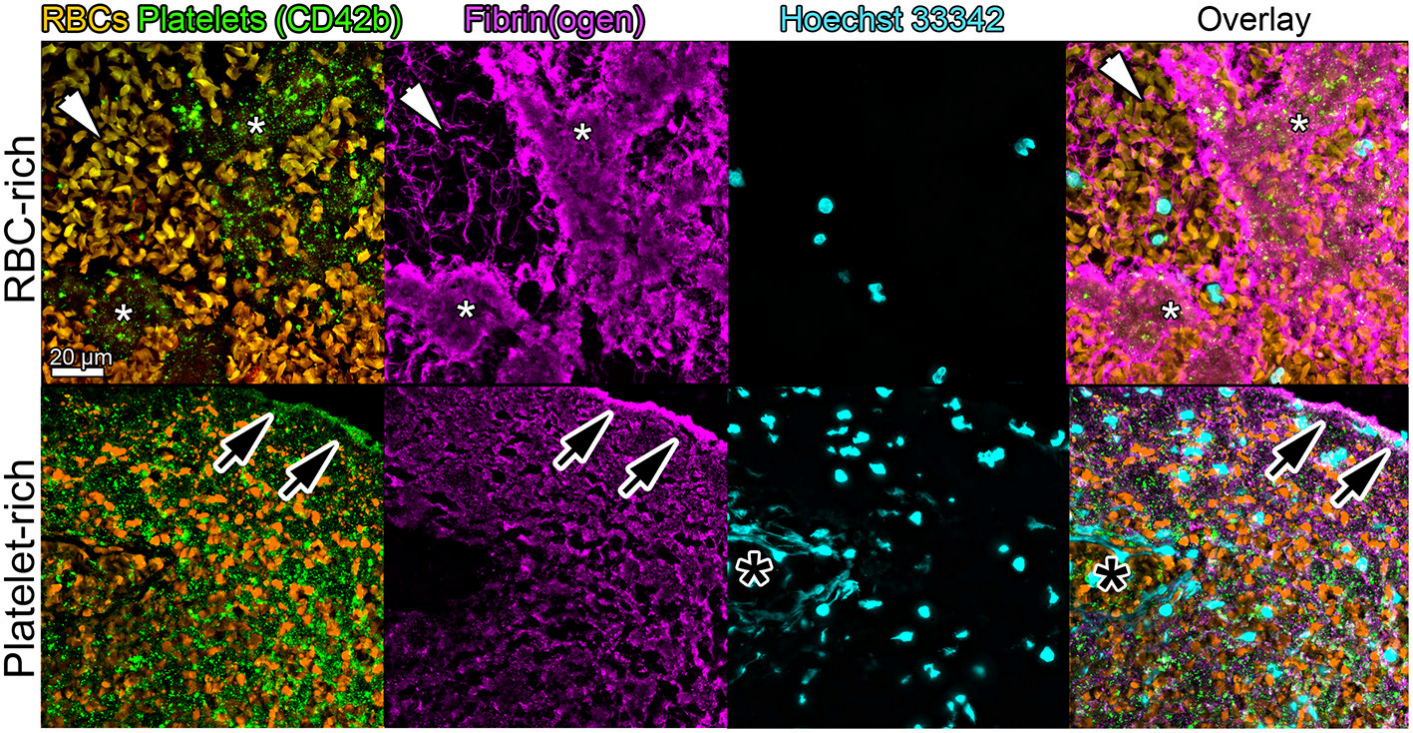
Immunohistological aspects of retrieved thrombi causing LVO in the simulating system. Representative images showing platelets (CD42b, green), red blood cells (RBCs, yellow), fibrin (ogen) (magenta), and DNA (Hoechst 33,342, blue) staining in the core of a retrieved RBC-rich (upper panels) or platelet-rich (lower panels) thrombus. Note the stark contrast in fibrin structure between the platelet-rich (*, white asterisks) and RBC-rich (white arrowheads) areas in the inner core of the RBC-rich thrombus. Also worth noting is the accumulation of fibrin and platelets at the thrombus periphery as shown by the black arrowheads in the platelet-rich thrombus images. The black asterisks indicate the presence of uncondensed DNA, suggestive of neutrophil extracellular traps.

Hematoxylin and Eosin staining showed that both RBC- and platelet-rich thrombi had a platelet- and fibrin-rich outer layer encapsulating a core in which RBCs were predominantly found. This external layer was thicker in platelet-rich thrombi compared to the RBC-rich thrombi (Figure 3C). Immunostaining for platelets, fibrin (ogen), and DNA confirmed this structural organization, with an external layer composed of a dense meshwork of platelets and fibrin (ogen), and a heterogeneous inner core made of RBC-rich regions with a thin and loose network of fibrin (ogen), and platelet-rich areas associated with densely compacted fibrin (ogen) (Figure 5).

## Discussion

In agreement with the recent studies using *in vitro* simulators for cerebral arterial EVT procedures (8, 9), we show here that such simulators could be instrumental understanding the impact of thrombus composition on EVT strategies. In fact, we show that RBC-rich thrombi are associated not only with faster stent retriever-mediated recanalization but also with more distal emboli, than the platelet-rich thrombi.

An increasing trend of RBC-rich thrombi to produce emboli during *in vitro*-simulated stent retriever-mediated recanalization was also reported by Madjidyar et al. who compared RBC-rich thrombi with fibrin-rich thrombi (8). These results are also in line with those of retrospective clinical studies (15–17). In particular, RBC-rich thrombi were shown to be associated with a higher probability of successful reperfusion (15), a shorter intervention time, and a better clinical outcome, as compared to fibrin/platelet-rich thrombi (16). Our results also strengthen the notion that thrombus composition is an important determinant for thrombus friction and elastic properties (18–20) and therefore potentially of EVT success. For example, fibrin-rich thrombi were previously reported to have higher friction coefficients than the RBC-rich thrombi (18) and to be associated with poorer EVT outcomes (21). Interestingly, our results indicate that, in addition to impairing thrombolysis (14, 22), platelets also make EVT more difficult.

Per-procedural thrombus fragmentation, which can lead to distal embolization with final incomplete reperfusion or, in worst cases, to embolization in a nonaffected territory (ENT) (23), has been reported to be associated with thrombus histology. The literature concerning the association between thrombus RBC concentration and thrombus fragmentation occurrence is controversial. Some clinical studies have reported that there was either a lower (21) or higher (24) risk in the fragmentation of RBC-rich thrombi. While our results indicate that increased RBC content is associated with an increased risk of thrombus fragmentation and distal embolization during EVT, our model could be used to address the impact of other cell types like leukocytes on thrombus fragility. Recent studies have indeed suggested that increased neutrophil content may be associated with a higher risk of fragmentation (25, 26).

The mechanisms underlying the negative impact of platelets on EVT remain to be investigated further but results from previous studies suggest that they may involve platelet-mediated modifications and compaction of fibrin and other thrombus components (9, 10, 14, 22). In this regard, it is worth noting that thrombi retrieved from the simulation system displayed structural features comparable to those previously observed in thrombi from AIS patients with LVO, with a peripheral outer layer characterized by the accumulation of platelets and densely matted fibrin (ogen), and a heterogeneous inner core made of RBC-rich and platelet-rich areas, respectively, associated with clearly distinguishable fibrin fibers and compacted fibrin (ogen) (14, 22). Extracellular DNA, which was previously reported to contribute to the resistance of AIS thrombi to intravenous thrombolysis (13, 27), was also found in the thrombi retrieved from the simulating system. Whether extracellular DNA affects EVT remains an open question that could be explored using *in vitro* simulators for large vessel occlusion.

The increased difficulty to retrieve platelet-rich thrombi is often attributed in part to contraction-associated compression of fibrin fibers and an increase in thrombus stiffness and friction (9, 18, 28). Recently, Johnson et al. showed that platelet-contracted thrombi were more difficult to retrieve than noncontracted thrombi after being injected in an experimental setting comparable to the one we used here (9). Strikingly, we observed that both RBC-rich and platelet-rich thrombi saw a drastic reduction in their size after being injected into the simulating model. This indicates that hydrodynamic pressure is a major determinant of thrombus compaction that adds to and may surpass the well-known effect of aggregated platelets on thrombus volume (9, 10, 14, 20, 22). It further suggests that compaction *per se* does not explain the increased resistance of platelet-rich thrombi to EVT and that the inherent adhesive properties of platelets might contribute to the increased friction of such thrombi. Considering the relevance of thrombus compaction in resistance to thrombolysis (22, 29–31), the fact that *in vitro* and in flow models incorporate this parameter reinforces their relevance to the pathophysiology of AIS.

There are several limitations to this study. First, all procedures were performed by only one experienced neuro interventionalist. Whereas, this might have been an asset in terms of reproducibility, considering that, to date, the experience of the neuro interventionalist plays a major role in EVT execution, replication of the results by different neuro-interventionalists would strengthen their generalizability. Furthermore, although RBC-rich and platelet-rich areas represent the main structural domain types in AIS thrombi (14), the two categories of thrombi studied here do not represent the entire spectrum of AIS thrombi. Also, one cannot exclude an impact of the thrombus storage conditions prior to embolization in the system on thrombus composition and EVT outcome. Nevertheless, both types of thrombi displayed remarkable features of AIS thrombi, including the presence of a compact outer layer, a heterogeneous core, and extracellular DNA strands. One can envision finely tuning the proportions of platelets, RBCs, or even extracellular DNA, by adjusting the composition of blood cell preparations prior to clotting initiation and thrombus injection. In addition, while we used the blood from healthy volunteers for generating thrombi, the use of blood from AIS patients or from subjects with vascular risk factors for AIS-like diabetes, high blood pressure, or atrial fibrillation may allow for the production of more clinically relevant thrombi. Further studies using different thrombus compositions and including AIS risk factors may help to better understand the mechanisms underlying the thrombus fragmentation or EVT resistance. Finally, the impact of thrombolysis on EVT is another parameter that could be explored in combination with that of thrombus composition using EVT simulation systems. Indeed, bridging therapy (i.e., intravenous thrombolysis (IVT) before EVT) remains the gold standard recanalization treatment for AIS (32–34), and results from a recent study suggest that IVT alters thrombus composition (35).

## Data Availability Statement

The original contributions presented in the study are included in the article/Supplementary Material, further inquiries can be directed to the corresponding author.

## Author Contributions

AF, J-PD, FD, MM, VO, BH-T-N, RB, and MP designed the study and collected and interpreted the data. AF, J-PD, and BH-T-N wrote the manuscript. J-PD performed the statistical analyses. All other authors collected and interpreted the data, and revised the manuscript.

## Funding

This work was supported by a personal legacy donation from Héla Salomon and by the French National Research Agency (ANR) as part of the Investments for the Future program (PIA) under grant agreement No. ANR-18-RHUS-0001 (RHU Booster) and ANR-16-RHUS-0004 (RHU TRT_cSVD).

## Conflict of Interest

The authors declare that the research was conducted in the absence of any commercial or financial relationships that could be construed as a potential conflict of interest.

## Publisher's Note

All claims expressed in this article are solely those of the authors and do not necessarily represent those of their affiliated organizations, or those of the publisher, the editors and the reviewers. Any product that may be evaluated in this article, or claim that may be made by its manufacturer, is not guaranteed or endorsed by the publisher.
