# Supplementary material for: Modeling Large Vessel Occlusion Stroke for the Evaluation of Endovascular Therapy According to Thrombus Composition
Source: Front Neurol. 2022 Jan 27;12:815814. doi: 10.3389/fneur.2021.815814 (PMC8829452; doi:10.3389/fneur.2021.815814)
Supplement: Supplementary file 1 [file Table_1.DOCX]

Supplementary Material

**Supplemental video legend.**

Representative video showing the injection and embolization of a thrombus leading to a large vessel occlusion of the M1 segment of the left MCA in the acute ischemic stroke simulation system.
